# Benign functional anorectal conditions: a multi-centre analysis of rectal stump symptomatology in patients undergoing stoma formation

**DOI:** 10.1007/s10151-025-03192-6

**Published:** 2026-03-27

**Authors:** S. Balaji, C. Clark, A. Warwick, C. Gillespie

**Affiliations:** 1 Department of Colorectal Surgery, Queen Elizabeth Jubilee II Hospital, Coopers Plains, 4108 Australia; 2 Faculty of Medicine, University of Queensland, Herston, 4006 Australia; 3 Department of Colorectal Surgery, Mater Hospital, South Brisbane, 4101 Australia

**Keywords:** Rectal stump, Stoma, Mucus, Functional anorectal

## Abstract

**Purpose:**

Functional anorectal conditions are common, and management is challenging. Conservative management may not always be successful and some of these patients may opt for stoma formation. Rectal stump symptomatology after surgery is poorly understood with limited existing literature in this field. The aim of this study is to investigate the prevalence of rectal stump symptomatology in patients undergoing stoma creation for benign functional anorectal conditions.

**Method:**

A retrospective chart review for patients undergoing stoma formation for functional anorectal conditions at two metropolitan hospitals was conducted. Patient demographics and incidence of rectal stump symptoms were documented and analysed.

**Results:**

Thirty-nine patients underwent stoma formation over a 7-year period. The average age was 55 years and 90% of patients were female. End colostomy was performed in 23 (59%) patients, one patient underwent a loop colostomy, and the remaining patients (15) underwent loop ileostomy. Post-operative symptoms associated with the rectal stump were common and included problematic rectal mucus discharge (51%), pelvic pain (21%), rectal impaction (5%), tenesmus (3%) and rectal prolapse (3%). Overall, 65% of patients underwent at least one intervention for persistent rectal mucus discharge, including enemas (38%), irrigation (38%) or completion proctectomy (23%). In total, 92% of patients were noted to have satisfactory control of symptoms with a median follow-up time of 3 years.

**Conclusions:**

Rectal stump symptomatology is common in patients undergoing a defunctioning stoma for benign functional anorectal conditions. Many of these patients require ongoing management to control their symptoms, including in some cases a completion proctectomy. This study forms the basis for further research on rectal stump symptomatology in this complex cohort of patients.

## Introduction

Benign functional anorectal conditions (BFAC) include functional constipation and faecal incontinence [1]. While these disorders are not life-threatening, they can significantly impact a patient’s quality of life [2]. Symptoms include obstructed defecation, leakage and faecal urgency, with many patients presenting with mixed symptoms.

The pathophysiology of BFAC encompasses disordered brain–gut axis, pelvic organ prolapse, neuropathy and sphincter deficiency [3]. Management options include conservative, medical and surgical approaches. Conservative treatments are defined as dietary changes, pelvic floor physiotherapy, psychology and biofeedback [4]. This is often used in conjunction with pharmacological therapies such as laxatives, loperamide, fibre supplementation and antispasmodics. Other measures include trans-anal or colonic irrigation. When these measures fail, surgery may be considered. Surgical options include sacral neuromodulation, sphincter repair, prolapse surgery and stoma formation [5]. Stoma formation is often utilised in those with intractable symptoms when other measures may have failed [6]. The proportion of patients with BFAC undergoing stoma formation is estimated to be 1% [7].

BFACs are highly prevalent in the wider community and have a reported prevalence between 17% and 36% [8]. Despite this, management of these conditions is challenging; conservative measures are not always successful and surgical intervention when utilised may also have poor efficacy [9]. Accordingly, ongoing management of patients with BFACs is often concentrated within specialised pelvic floor centres [10]. If stoma formation is deemed appropriate, the options include an ileostomy or colostomy. While studies have shown patient satisfaction can be high with a stoma [11], the defunctioned bowel left in situ can lead to discomfort or other problems in these patients. This may be related to persisting pelvic floor dysfunction obstructing the rectal stump or causing spasm, or persisting leakage of the mucus secreted by the bowel left in continuity. Occasionally, accumulation of inspissated mucus may lead to impaction or pressure on the anus. Symptoms can occur early after stoma formation and include tenesmus, mucus discharge, urgency, pain, excoriation and bleeding. There is limited published literature reporting symptoms from the defunctioned rectal stump in patients with BFAC. Rather, much of the current literature on the retained rectal stump comes from the inflammatory bowel population, which has limited applicability to the BFAC population. In this context, a recent study demonstrated that 100% of patients suffer inflammation related to their rectal stump; this may be indicative of persisting inflammation causing early symptoms, or diversion proctocolitis causing symptoms years later [12, 13]. Davis et al. [14] published a retrospective study examining both surgical and patient reported outcomes for a cohort of patients with severe chronic constipation who underwent an ileostomy. This study demonstrated that 51% of patients experienced some form of regret undergoing the surgery, with 24% experiencing higher levels. The reasons for the regret are likely multifactorial; however, the authors mention that rectal mucus discharge may be a contributing factor.

Counselling patients on stoma formation in the BFAC population remains challenging as there is limited literature that can guide both patients and clinicians on expected symptoms from the defunctioned rectal stump. Symptoms relating to the rectal stump may significantly impact on the patient’s quality of life post-operatively. The aim of this study is to investigate the prevalence of rectal stump symptomatology and other post-operative complications in patients undergoing stoma creation for benign functional anorectal conditions.

## Materials and methods

### Ethics

Ethics approval was granted by the Metro South Hospital and Health Service Human Research Ethics Committee and Mater Hospital.

### Study design

A retrospective study using data from patients who have undergone stoma formation for pelvic floor dysfunction between 2016 and 2023 at both Queen Elizabeth Jubilee II Hospital and Mater Private Hospital South Brisbane was conducted. All patients who had undergone stoma formation within these institutions were identified using search parameters within the electronic medical records.

A low-risk ethics application was approved by the Metro South Hospital and Health Service. Only patients who had undergone stoma formation for a benign functional bowel disorder were included. Exclusion criteria were defined as patients who had undergone stoma formation with no distal bowel remaining and those with incomplete follow-up medical records.

Medical records were reviewed and data collected included demographics, reason for stoma, ileostomy or colostomy formation, length of follow-up, post-operative complications, rectal symptoms, medical comorbidity, treatment for rectal stump symptoms, satisfaction with treatment and endoscopic assessment.

### Data analysis and statistics

Statistics were generated using IBM SPSS version 26. Descriptive statistics were used to characterise the study demographics. Continuous date was summarised using median and interquartile range (IQR).

## Results

Thirty-nine patients were identified to have suffered from BFAC and subsequently underwent stoma formation during the selected time period. Overall, 90% of patients were female (*n* = 35), the rest were male (*n* = 4). The average age was 55 years with a range of 20–87 years. Nineteen patients (49%) had comorbidities. The comorbidities included: heart disease, physical disability, obesity, obstructive lung conditions, depression/anxiety and post-traumatic stress disorder.

**Fig. 1:**
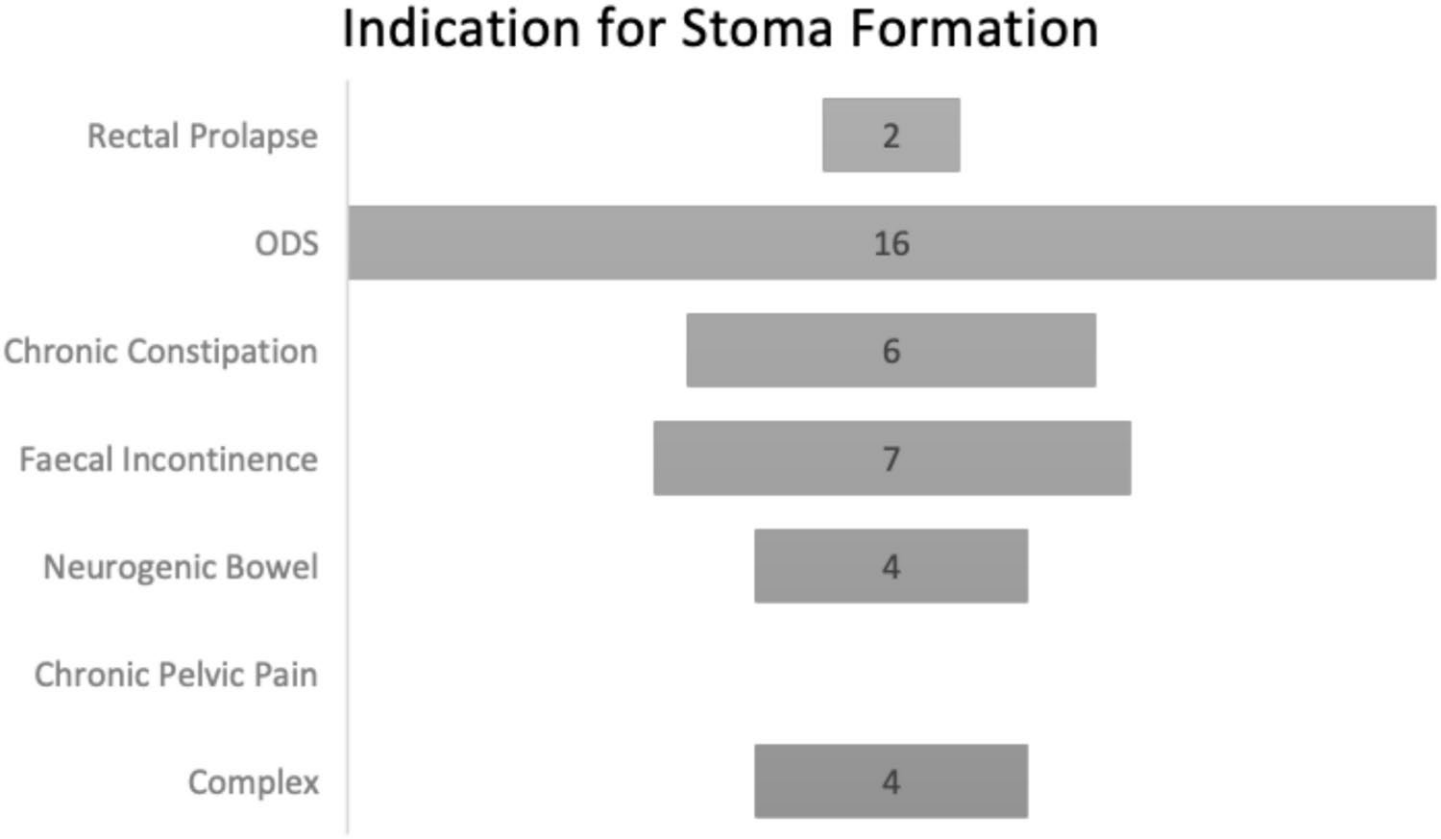
Indication for Stoma Formation

Stoma formation was utilised in patients with intractable symptoms. The most common indication for stoma formation was severe obstructive defecation syndrome (ODS) (41% [*n* = 16]). Other common indications included: faecal incontinence (18%), slow-transit constipation (15%), neurogenic bowel (10%) and rectal prolapse (5%). Overall, 10% of patients suffered from more than one indication. Such patients were defined as having a complex indication for stoma formation. These data can be seen represented in Fig. 1. The most common procedure performed was an end colostomy (59% of patients), followed by 38% of patients who underwent a loop ileostomy and 3% who underwent a loop colostomy.

Operative outcomes can be seen in Table 1. Overall, 36% of patients experienced stoma related complications. These complications included development of a parastomal hernia (*n* = 9), stomal prolapse (*n* = 2), stomal stenosis (*n* = 2), stoma retraction and small bowel obstruction (*n* = 1). In total, 51% of patients suffered from problematic rectal mucus discharge following formation of a defunctioning stoma. This was more common in those undergoing a loop ileostomy (60%). Pelvic pain (21%), rectal impaction (5%), tenesmus (3%) and rectal prolapse (3%) were other common symptoms. Overall, 12 out of 23 patients who received an end colostomy experienced rectal stump symptoms (52%). In contrast, 10 out of the 15 patients who received a loop ileostomy experienced rectal stump symptoms (67%). The single patient to receive a loop colostomy experienced a rectal prolapse. All patients undergoing a loop ileostomy or loop colostomy experienced complications overall, either stoma related complications or rectal stump symptoms. Only 3% of patients underwent rectal stump endoscopic assessment.

**Table 1:** Outcomes

Outcomes	End colostomy (*n* = 23)	Loop ileostomy (*n* = 15)	Loop colostomy (*n* = 1)	Total (*n* = 39)
Stoma related complications				
Yes	9	4	1	14
No	14	11	0	25
Rectal stump symptoms				
Pelvic pain	4	4	0	8
Mucus discharge	11	9	0	20
Tenesmus	1	0	0	1
Rectal impaction	0	2	0	2
Rectal prolapse	0	0	1	1
No symptoms	11	0	0	11

There was a high incidence of mucus discharge in the cohort; this was problematic and bothered many patients, leading to intervention in 65% of patients. Table 2 outlines the management of mucus discharge. In those who had a loop ileostomy, all patients with mucus discharge underwent intervention. Intervention included trans-anal irrigation, rectal suppositories or disimpaction of mucus in the operating theatre. Three patients underwent further surgery in the form of completion proctectomy for management of mucus discharge. Intervention was most successful in the loop ileostomy group, with 78% having improvement in symptoms compared with only 18% of those with an end colostomy. Success was defined as patients being satisfied with the treatment given to manage the rectal discharge as documented in the notes. It should be noted that the single patient who underwent a loop colostomy did not experience mucus discharge.

**Table 2:** Management of Mucus Discharge

Management of Mucus Discharge	End Colostomy (*n* = 11)	Loop Ileostomy (*n* = 9)	Total (*n* = 20)
Intervention			
Yes	4 (36%)	9 (100%)	13 (65%)
No	7 (64%)	0	7 (35%)
Success of Intervention			
Yes	2 (50%)	7 (78%)	9 (45%)
No	2 (50%)	1 (11%)	10 (50%)
Unknown	0	1 (11%)	1 (5%)

Table 3 demonstrates the long-term outcomes. Reversal of defunctioning stoma was performed in two patients with end colostomies as the patients developed recurrent parastomal hernia. Overall, 92% of patients were successfully discharged from the care of the respective treating teams following satisfactory control of symptoms. One patient died during the follow-up period (unrelated to stoma) and one patient was lost to follow-up.

**Table 3:**
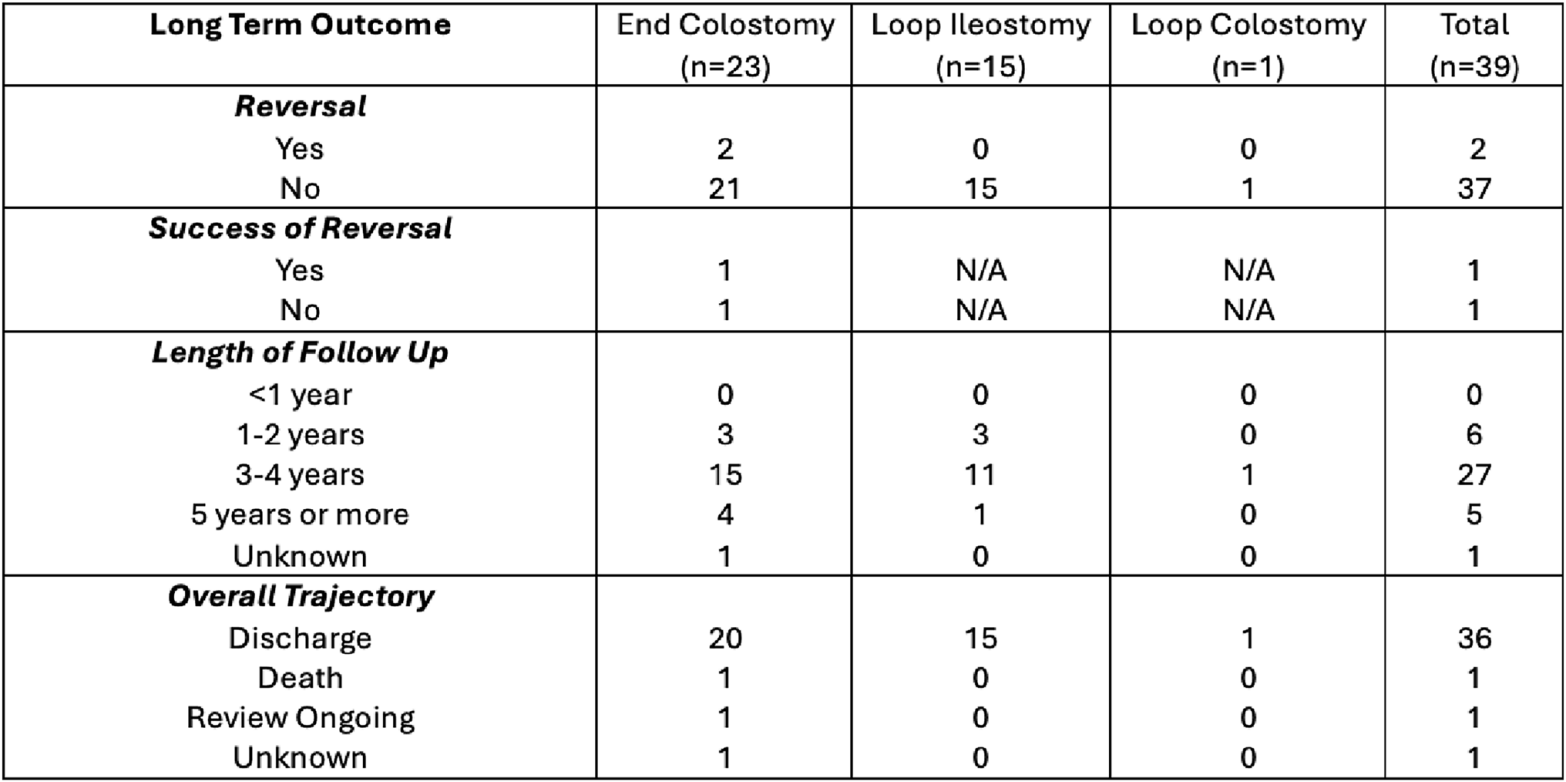
Long-term outcomes

Table 4 demonstrates that symptoms were broadly similar between the group subtypes; however, it appeared that faecal incontinence and chronic constipation groups had the highest rates of rectal mucus discharge. Greater numbers would be required to detect statistically significant differences.

**Table 4:** Proportion of patients with rectal mucus discharge based on indication

Indication for stoma	Proportion of patients with rectal mucus discharge
Defaecatory disorder	6/15 (50%)
Chronic constipation	6/9 (60%)
Faecal incontinence	6/10 (60%)
Other	2/5 (40%)

## Discussion

This study demonstrates that a significant number of patients who undergo a defunctioning stoma for BFAC have symptoms related to their rectal stump. In this review of 39 patients, we found that 51% have symptoms related to their rectal stump overall, and in the majority these symptoms were significant enough for them to undergo intervention. All patients with a loop ileostomy developed complications in this small series. This may suggest that a longer length of defunctioned colorectum predisposes for more symptoms related to mucus. The most common symptom reported was mucus discharge, followed by pelvic pain. It should be noted that the proportion of patients who had pain prior to stoma formation could not be definitively identified. Intervention for mucus discharge was undertaken in 65% of cases. These included conservative measures such as enemas and trans-anal irrigation. In two severe cases, patients underwent an abdominoperineal resection to excise the rectal stump. Overall, the treatment strategies were successful in only 66% of cases, demonstrating the challenges managing this issue.

While the numbers were small, this study demonstrated that patients with an end colostomy were less likely to experience rectal mucus discharge than those with a loop ileostomy. Interestingly only 36% of patients in the end colostomy group required treatment for rectal mucus discharge compared with 100% in the loop ileostomy group suggesting that mucus discharge may be less prominent for these patients. Treatment also appeared to be less successful in end colostomy group. This could be attributed to differences in the composition of mucus discharge that forms following an end colostomy, where it is more resistant to evacuation with enemas/irrigation. Common types of enemas used included microlax, steroid and sucralfate enemas. Owing to the retrospective nature of this study, the type of enemas/irrigation and frequency of usage was not always available. Determining these factors would be important in defining treatment response and highlights the importance of rigorous documentation for improving the quality of research into this area. In this study only 3% of patients underwent endoscopic assessment of the rectal stump – the length of the rectal stump may be another important characteristic that may impact on symptoms but was not assessed in this paper.

No patients in this study had undergone prior sphincter preserving rectal surgery or radiation treatment, which is important to mention as low anterior resection syndrome (LARS) may also present with severe functional symptoms that require stoma formation [15, 16]. The treatment and symptoms may vary to those patients who have not undergone prior treatment, and this cohort of patients is also worth considering.

To our knowledge, this is one of few studies evaluating symptoms related to the rectal stump in defunctioned patients with BFAC. Despite this, there were several limitations noted in this study. Firstly, the retrospective nature of this study introduces bias and potential inaccuracies regarding the recording of exact treatment response. Further, the small sample size reduces the power of the study and makes it difficult to draw conclusions, despite clear demonstration of clinical significance. Owing to the small sample size, only descriptive statistics were performed. A larger sample size would allow inferential statistical analysis and more statistically significant conclusions to be made. Nonetheless, it is acknowledged that the current research base on BFAC and its management is limited, and this study forms the basis for further research. The multicentre nature of this study adds to the strength of this paper; however, expanding to other sites or even the formation of a national database may be required to collate data on a larger cohort of patients owing to the rarity of these operations in this clinical scenario.

These patients often suffer from a lack of resources and are a heterogeneous group, which makes them difficult to study. The care they receive is often fragmented, from different specialists and institutions, which may explain why this topic of study has not been researched in detail. At the institutions that this study was conducted in, the facilities host a specialist functional anorectal unit allowing close follow-up of such patients. In the experience of surgeons who work in this unit, the rectal stump is a frequent source of dissatisfaction for these patients, and this is clearly reflected in the results of this study. It is also worth noting that 39% of patients suffered a stoma related complication, such as a hernia or prolapse. Both rectal stump symptoms and these stoma-related complications predispose patients to a high rate of further intervention following stoma formation.

## Conclusions

Within the limitations discussed, this study demonstrates that both rectal stump symptomatology and stoma-related complications are common in patients undergoing a defunctioning stoma for BFAC. Many of these patients require ongoing management to control their symptoms, including in some cases a completion proctectomy. This study lays the foundation for further prospective study into this important topic, both its aetiology and management.

## Data Availability

No datasets were generated or analysed during the current study.
